# RORα suppresses interleukin-6-mediated hepatic acute phase response

**DOI:** 10.1038/s41598-019-48171-8

**Published:** 2019-08-13

**Authors:** Ju-Yeon Kim, Yong-Hyun Han, Min-Woo Nam, Hyeon-Ji Kim, Mi-Ock Lee

**Affiliations:** College of Pharmacy and Bio-MAX institute, Research Institute of Pharmaceutical Sciences, Seoul National University, Seoul, 08826 Republic of Korea

**Keywords:** Molecular biology, Transcription, Cell biology

## Abstract

Acute liver failure (ALF) is characterized by loss of liver function in response to sustained augmentation of the acute-phase response (APR) in the liver, which can progress even to death. Although the inflammatory interleukin-6 (IL-6)–axis is a crucial factor that drives the hepatic APR by releasing diverse acute-phase proteins (APPs), therapeutic strategies to block the IL-6–STAT3-mediated APR are not well developed. Here, we show that the nuclear receptor retinoic acid-related orphan receptor α (RORα) limits APR-mediated liver injury by inhibiting the hepatic IL-6–STAT3 signaling pathway. Administration of JC1-40, an RORα activator, diminished diethylnitrosamine-induced acute liver injury and repressed transcriptional expression of APPs such as CXCL1 and LCN2 in mice. IL-6-mediated activation of STAT3 was repressed after RORα activation by either adenoviral infusion of RORα or JC1-40 treatment in primary hepatocytes. Activation of RORα decreased transcriptional expression of IL-6 receptor α, an upstream activator of STAT3, both *in vitro* and *in vivo*. This may be one mechanism underlying the RORα-mediated inhibition of STAT3. Taken together, our results suggest that RORα is a regulator of the hepatic IL-6–STAT3 signaling pathway and may be a new therapeutic target for treating APR-associated inflammatory ALF.

## Introduction

Acute liver failure (ALF) is a life-threatening disease caused by severely impaired hepatic function and can lead to coagulopathy, encephalopathy, systemic inflammatory response syndrome, multiorgan dysfunction, and even death^1^. The main cause of ALF is drug-induced hepatic injury in developed countries and viral hepatitis in developing countries. Autoimmune hepatitis and ischemic injury are also known to trigger ALF^1^. At present, therapeutic options for patients with ALF are limited, and the only recommendation is liver transplantation^2^.

The acute-phase response (APR) is a core mediator of local tissue injury-induced innate immunity^3^. The APR is initiated by an elevated level of proinflammatory cytokines, such as interleukin 6 (IL-6), which drive production of diverse plasma proteins called acute-phase proteins (APPs). APPs include C-reactive protein (CRP), plasminogen activator inhibitor 1 (PAI1), lipocalin 2 (LCN2), and complements^4^. APPs are synthesized primarily in hepatocytes and contribute to innate immunity through their functions in coagulation, fibrinolysis, antiproteases, and inflammation^5,6^. A transient activation of the APR is involved in the defense against infection and tissue restoration, but a sustained APR can amplify the inflammatory response, which may cause tissue injury. Liver X receptor, liver receptor homolog-1, and peroxisome proliferator-activated receptor alpha have been identified as negative regulators of the APR that prevent sustained inflammation^7^.

IL-6 is secreted by Kupffer cells, monocytes, and T cells, and is the major mediator of the APR that induces the transcriptional expression of APPs in the liver. Binding of IL-6 to IL-6 receptor α (IL-6Rα) elicits formation of a complex with glycoprotein 130, which in turn activates Janus kinase (JAK) followed by activation of signal transducer and activator of transcription 3 (STAT3)^8,9^. Phosphorylation at tyrosine 705 (pSTAT3) activates STAT3, which leads to dimerization, entry into the nucleus, and subsequent induction of downstream target genes^9^.

There is strong evidence that the IL-6–STAT3 pathway is important in the regulation of APR genes. For example, the APR is impeded in IL-6-knockout or hepatocyte-specific IL-6R-knockout mice^10–12^. In addition, STAT3 binding to the promoters of APR genes, such as α2-macroglobulin, is absent in IL-6-knockout mice^13^. Therefore, the IL-6–STAT3 pathway is considered to be an ideal target for the control of the APR; however, effective therapeutic interventions to inhibit this pathway have not been identified.

The orphan nuclear receptor, retinoic acid-related orphan receptor α (RORα), is a ligand-dependent transcriptional factor that regulates diverse target genes associated with hepatic inflammation and damage^14^. RORα attenuates the proinflammatory response, including secretion of IL-6 in smooth muscle cells, by negatively regulating NF-κB activation^15^. Further, RORα plays a pluripotent role in the bidirectional control of IL-6 signaling, including direct activation via binding to the IL-6 promoter and indirect suppression via inhibition of NF-κB signaling in astrocytes^16^.

We recently reported that hepatic expression of IL-6 was much higher after high-fat diet feeding in myeloid-specific RORα-knockout mice than in control mice^17^. We have also shown that RORα protects against liver damage and reduces hepatic inflammation^17–19^. Here, we report that RORα suppresses the hepatic APR by disrupting hepatic IL-6–STAT3 signaling. Further, we identified a mechanism by which RORα reduces the transcriptional expression of IL-6Rα in hepatocytes.

## Results

### Administration of the RORα activator, JC1-40, reduces diethylnitrosamine-induced APR in the liver

**Figure 1:**
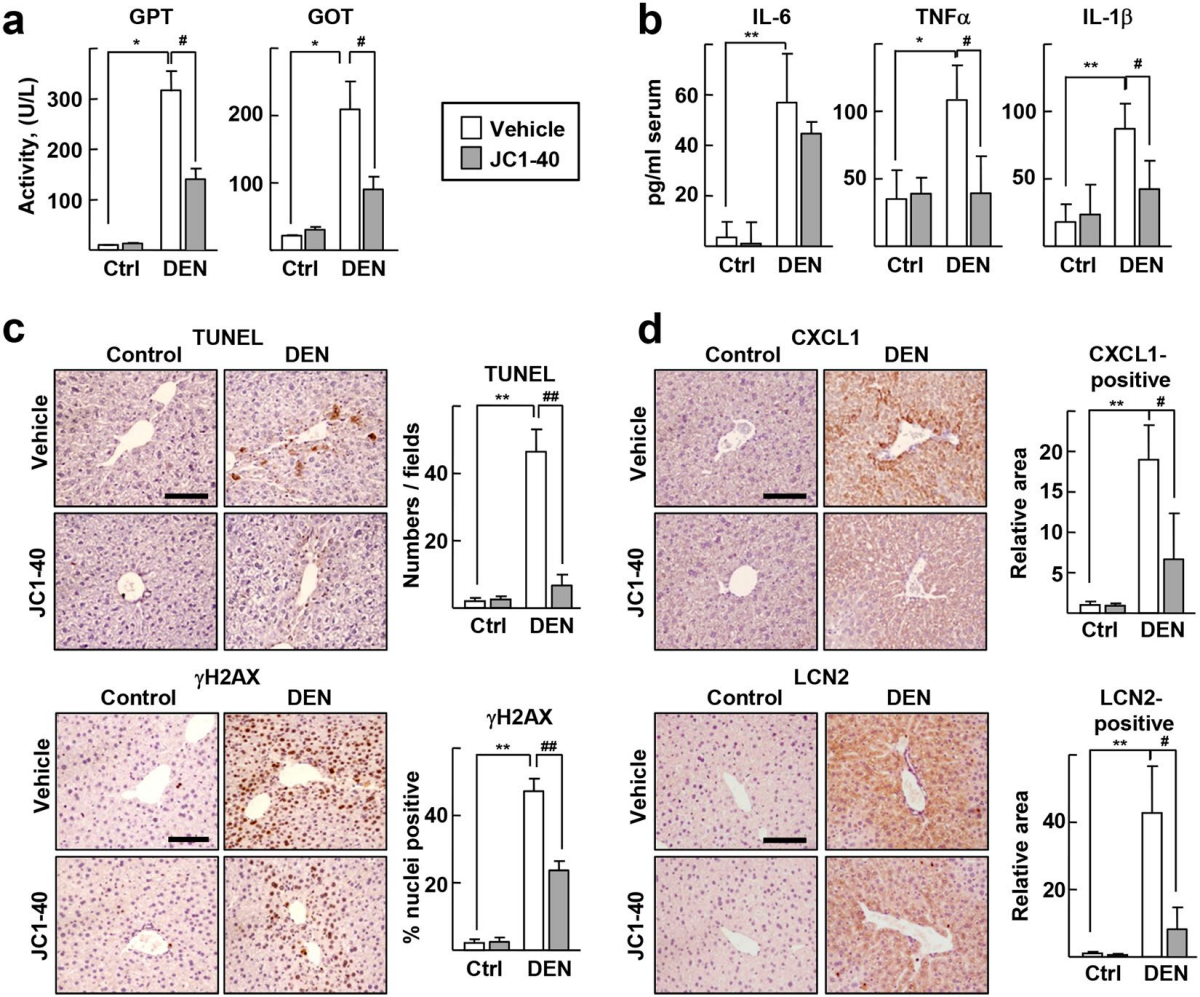
Administration of JC1-40 reduces the DEN-induced APR in the liver. JC1-40, 20 mg/kg BW/day, was administered orally for 3 days to the mice and then the mice were i.p. injected with 100 mg/kg BW DEN for 2 days before sacrificed. (**a**) Serum GPT and GOT activity were measured. (**b**) Serum levels of IL-6, TNFα, and IL-1β were determined by ELISA. (**c**) TUNEL staining of liver sections were conducted. TUNEL positive cells were counted using image J (top). Immunohistochemistry staining of γH2AX in the liver sections. The percentage of γH2AX positive nuclei was measured by Image J (bottom). (**d**) Immunohistochemistry staining of CXCL1 and LCN2 in the liver sections. The stained area were measured by Image J. The analysis of stainings were conducted from at least 6 images per tissues. Scale bars: 100 μm. *P < 0.05, and **P < 0.01; ^#^*P* < 0.05, and ^##^*P* < 0.01 (n = 3-5) for (**a**–**d**). The data represent mean ± SD.

To examine the role of RORα in modulating the hepatic APR, we used diethylnitrosamine (DEN), a liver toxin that induces acute liver injury and a proinflammatory response by promoting production of IL-6 in Kupffer cells in the mouse liver^20^. First, levels of serum glutamic pyruvic transaminase (GPT) and glutamic oxaloacetate transaminase (GOT), indicators of liver injury, increased significantly after DEN treatment, whereas coadministration of JC1-40, an RORα activator, decreased the levels of these markers (Fig. 1a). As expected, the serum IL-6 level was significantly increased by DEN treatment but was not affected by JC1-40 treatment. However, JC1-40 markedly decreased serum levels of the proinflammatory cytokines tumor necrosis factor α (TNFα) and IL-1β, which suggests that JC1-40 suppressed the proinflammatory response without reducing the IL-6 level (Fig. 1b). Consistently, the administration of JC1-40 attenuated DEN-induced signs of liver damage such as apoptotic cell death and DNA damage in the periportal region (Fig. 1c). In addition, JC1-40 decreased the number of infiltrating inflammatory leukocytes after DEN treatment (Supplementary Fig. S1). Immunostaining for representative APPs chemokine (C-X-C motif) ligand 1 (CXCL1) and LCN2 proteins showed that JC1-40 decreased the levels of these APPs (Fig. 1d). Taken together, these results suggest that JC1-40 suppressed the DEN-induced proinflammatory responses and hepatic APR.

**Figure 2:**
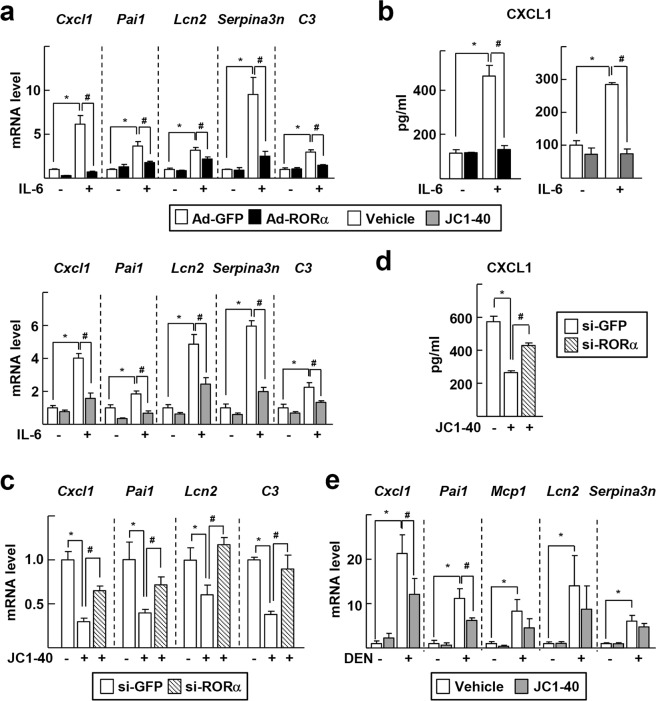
RORα regulates gene expression of APPs. (**a**,**b**) Primary mouse hepatocytes were infected by Ad-GFP or Ad-RORα for 24 h and then treated with 20 ng/ml IL-6 for 24 h (top). Or primary mouse hepatocytes were treated with 50 μM JC1-40 for 24 h in the presence or absence of 20 ng/ml IL-6 for 24 h (bottom). The mRNA levels of APR markers such as *Cxcl1*, *Pai1*, *Lcn2*, *Serpina3n*, and *C3* were analyzed by qRT-PCR (**a**). Culture media were collected and the level of CXCL1 was detected by ELISA (**b**). (**c**,**d**) Primary mouse hepatocytes were transfected with si-GFP or si-RORα for 24 h and then treated with 50 μM JC1-40 for additional 24 h in the presence of 20 ng/ml IL-6. The mRNA levels of APR markers such as *Cxcl1*, *Pai1*, *Lcn2*, *Serpina3n*, and *C3* were analyzed by qRT-PCR (**c**). Culture media were collected and the level of CXCL1 was detected by ELISA (**d**). (**e**) JC1-40, 20 mg/kg BW/day, was administered orally for 3 days to the mice and then the mice were i.p. injected with 100 mg/kg BW DEN for 2 days before sacrificed. The hepatic mRNA levels of APR markers were analyzed by qRT-PCR. *P < 0.05; ^#^*P* < 0.05 (n = 3) for (**a**–**d**) and (n = 3–5) for (**e**). The data represent mean ± SD. Representatives of at least three independent experiments with similar results are shown.

To examine whether RORα regulates the expression of APR genes, adenovirus encoding RORα, Ad-RORα, was infused into primary hepatocyte cultures. IL-6 treatment induced large increases in the mRNA levels of APR genes such as *Cxcl1*, *Pai1*, *Lcn2*, serine protease inhibitor A3N (*Serpina3n*), and complement component 3 (*C3*). However, Ad-RORα infusion decreased the mRNA levels of these genes. Similarly, treatment with JC1-40 suppressed the induction of APR genes (Fig. 2a). Consistently, the amount of secreted CXCL1 protein from IL-6-treated hepatocytes was reduced by either infusion of Ad-RORα or treatment with JC1-40 (Fig. 2b). JC1-40 induced a less or no inhibition of the APR gene transcription and CXCL1 secretion when RORα was knocked down, supporting the RORα-mediated JC1-40 effect (Supplementary Fig. S2 and Fig. 2c,d). Of the three ROR subfamily members, JC1-40 induced transcriptional activity of RORα in a dose-dependent manner, but did not induce that of RORβ and RORγ, suggesting that RORα mediated the effect of JC1-40 (Supplementary Fig. S3). Knockdown of RORα in the presence of IL-6 further enhanced the level of some APR genes, such as *Cxcl1* and *C3*, suggesting the involvement of RORα in the induction of these genes (Supplementary Fig. S4). As shown in Fig. 1, DEN treatment increased the hepatic mRNA levels of APR genes in mice, but treatment with JC1-40 decreased the level of these genes by 35–45% (Fig. 2e).

### RORα suppresses the IL-6 induced activation of STAT3 *in vivo* and *in vitro*

**Figure 3:**
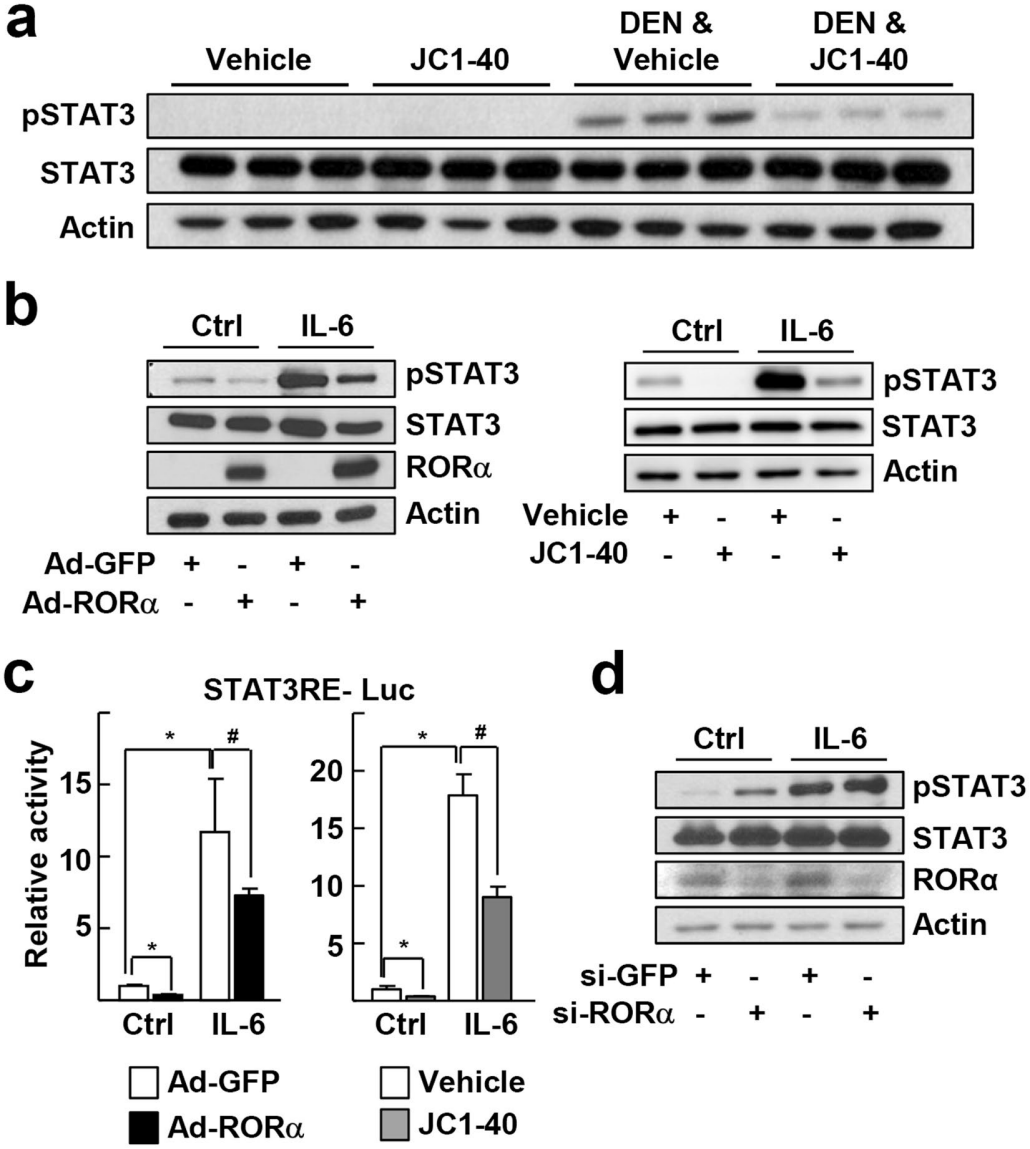
RORα represses IL-6-induced activation of STAT3. (**a**) JC1-40, 20 mg/kg BW/day, was administered orally for 3 days to the mice and then the mice were i.p. injected with 100 mg/kg BW DEN for 2 days before sacrificed. The protein levels of pSTAT3 in liver tissues were analyzed by western blotting. (**b**) Primary mouse hepatocytes were infected by Ad-GFP or Ad-RORα for 24 h and then treated with 10 ng/ml IL-6 for an additional 24 h (left). Or primary mouse hepatocytes were treated with 50 μM JC1-40 for 8 h in the presence or absence of 10 ng/ml IL-6 for 8 h (right). The protein levels of pSTAT3 and RORα in hepatocytes were analyzed by western blotting. (**c**) HepG2 cells were transfected with the STAT3RE-Luc reporter. After 24 h of transfection, cells were infected by Ad-GFP or Ad-RORα for 24 h in the presence or absence of 1 ng/ml IL-6 for 6 h (left), or treated with 50 μM JC1-40 for 24 h in the presence or absence of 1 ng/ml IL-6 for 6 h (right). Luciferase activities were normalized by corresponding β-galactosidase activity. *P < 0.05; ^#^*P* < 0.05 (n = 3). (**d**) Primary mouse hepatocytes were transfected with si-GFP or si-RORα for 24 h and then treated with 10 ng/ml IL-6 for an additional 24 h. The protein levels of pSTAT3 and RORα in hepatocytes were analyzed by western blotting. The data represent mean ± SD. Representatives of at least three independent experiments with similar results are shown.

We then explored whether RORα can suppress STAT3 activity, given that STAT3 is the major transcriptional regulator of APR gene expression. Administration of JC1-40 in mice attenuated the DEN-induced increase in pSTAT3 levels (Fig. 3a). Treatment of primary culture of hepatocytes with IL-6, a well-known inducer of STAT3, increased pSTAT3 levels markedly. By contrast, infusion of Ad-RORα or treatment with JC1-40 decreased both basal and IL-6-induced pSTAT3 levels (Fig. 3b). To confirm this inhibitory effect, we used a luciferase reporter gene encoding the STAT3 response element. Treatment of HepG2 cells with IL-6 increased the reporter activity, but the expression of RORα or JC1-40 treatment repressed the IL-6-induced STAT3 activity (Fig. 3c). The suppressive effect of JC1-40 was comparable with that of a STAT3 inhibitor, S3I-201 (Supplementary Fig. S5a)^21^. A combination of RORα overexpression and JC1-40 treatment achieved additional repression to compare with the single treatment (Supplementary Fig. S5b). Inversely, knockdown of RORα increased the level of pSTAT3 (Fig. 3d).

### RORα attenuates phosphorylation of STAT3 via decreasing transcriptional expression of IL-6Rα

**Figure 4:**
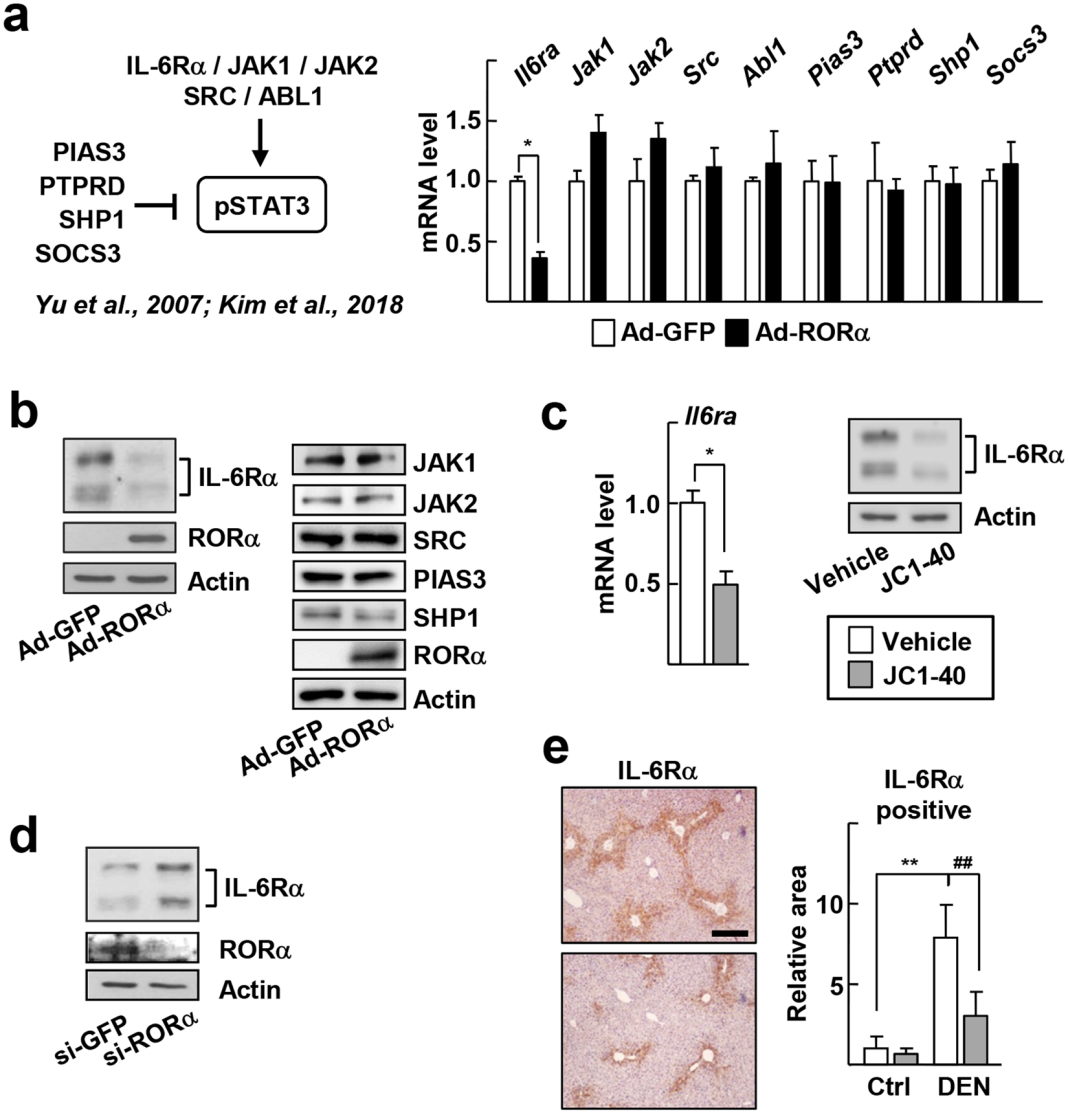
RORα represses expression of IL-6Rα. (**a**) A scheme for regulation of intracellular STAT3 signaling (left). Primary mouse hepatocytes were infected by Ad-GFP or Ad-RORα for 24 h. The mRNA levels of the indicated genes were measured by qRT-PCR (right). *P < 0.05 (n = 3). (**b**) Primary mouse were infected by Ad-GFP or Ad-RORα for 24 h. The protein levels of the indicated genes were analyzed by western blotting. (**c**) Primary mouse hepatocytes were treated with 50 μM JC1-40 for 24 h. The hepatic mRNA levels of IL-6Rα were analyzed by qRT-PCR (left). The protein levels of IL-6Rα and RORα in hepatocytes were analyzed by western blotting (right). *P < 0.05 (n = 3). (**d**) Primary mouse hepatocytes were transfected with si-GFP or si-RORα for 48 h. The protein levels of IL-6Rα and RORα in hepatocytes were analyzed by western blotting. (**e**) JC1-40, 20 mg/kg BW/day, was administered orally for 3 days to the mice and then the mice were i.p. injected with 100 mg/kg BW DEN for 2 days before sacrificed. Immunohistochemistry staining of IL-6Rα in the liver sections. The stained area were measured by Image J. Scale bars: 200 μm. **P < 0.01 and ^##^*P* < 0.01 (n = 5). The data represent mean ± SD.

**Figure 5:**
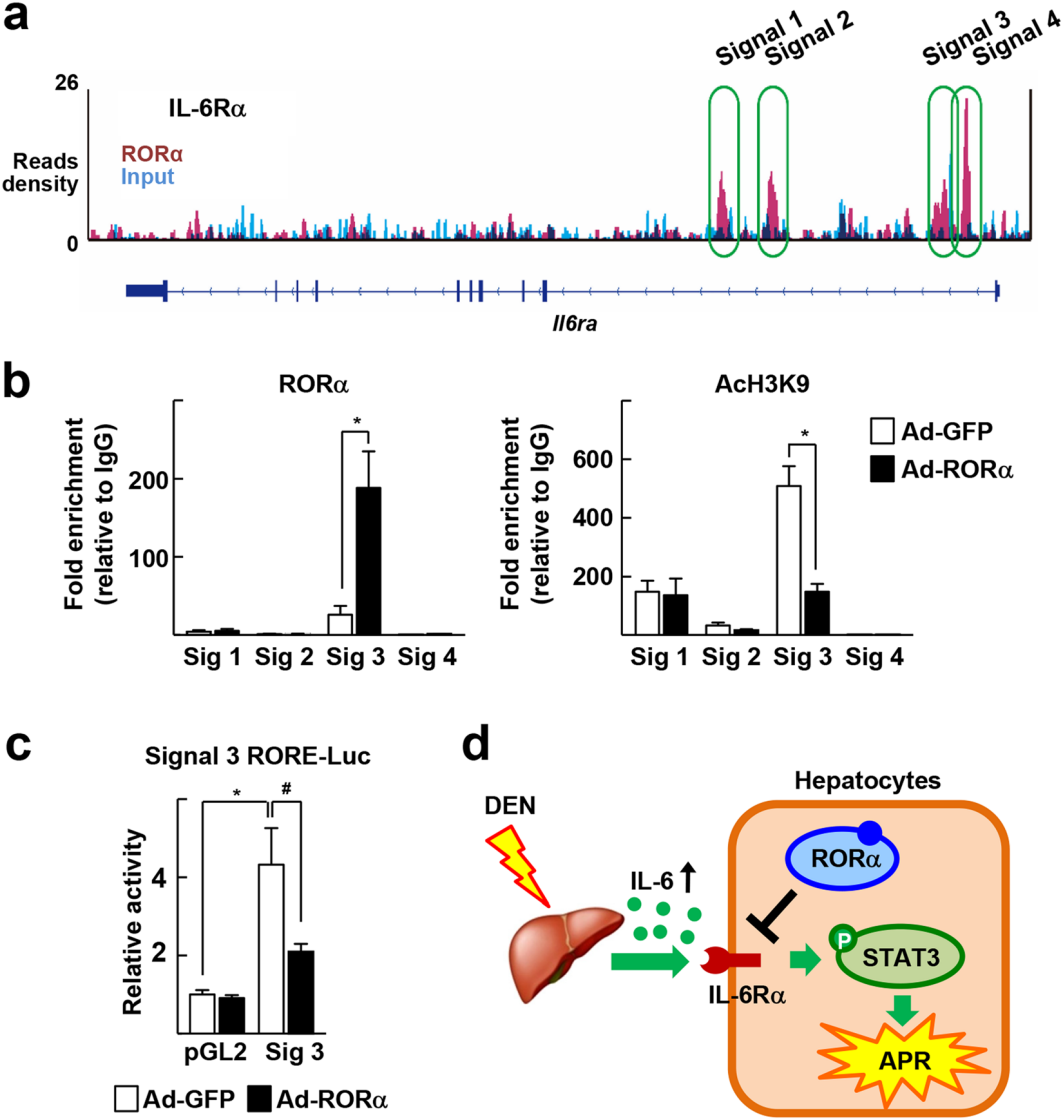
RORα suppresses transcription of IL-6Rα via direct binding to an intronic region of the mouse *Il6ra* gene. (**a)** ChIP-seq reads of RORα and liver input control in the genome loci of *Il6ra* are shown in ChIP-seq track. ChIP-seq peaks were visualized in Integrative Genomics Viewer (Broad Institute). (**b**) Primary mouse hepatocytes were infected by Ad-GFP or Ad-RORα for 24 h. DNA fragments that contain RORα enrichment region indicated by public ChIP-seq datasets were immunoprecipitated with the anti-RORα or anti-AcH3K9. (**c**) HepG2 cells were transfected with the Signal 3 RORE-luc reporter, and were infected by Ad-GFP or Ad-RORα for 24 h. Luciferase activities were normalized by corresponding β-galactosidase activity. *P < 0.05; ^#^*P* < 0.05 (n = 3). The data represent mean ± SD. (**d**) Schematic model for the suppressive mechanism of RORα in hepatic IL-6-STAT3-mediated APR.

To investigate further the mechanism responsible for the regulation by RORα of STAT3 activity, we examined whether RORα affects the expression of regulatory factors for STAT3 activity, such as IL-6Rα, JAK1, JAK2, proto-oncogene tyrosine-protein kinase Src (SRC), abelson murine leukemia viral oncogene homolog 1 (ABL1), protein inhibitor of activated STAT3 (PIAS3), protein tyrosine phosphatase, receptor type, D (PTPRD), and src homology 2 domain-containing phosphatase-1 (SHP1), and suppressor of cytokine signaling 3 (SOCS3) (Fig. 4a)^22,23^. Viral transduction of RORα decreased the mRNA and protein levels of *Il6ra* but not those of other proteins and mRNAs in hepatocytes (Fig. 4a,b). Also, treatment of JC1-40 decreased the expression of IL-6Rα at both mRNA- and protein-level in hepatocytes (Fig. 4c). In contrast, expression of IL-6Rα increased when RORα was knocked down (Fig. 4d). The DEN-induced expression of IL-6Rα in mouse liver was reduced markedly by coadministration of JC1-40 (Fig. 4e). Taken together, these results suggest that JC1-40 suppresses IL-6-mediated APR injury probably by decreasing expression of IL-6Rα. Data from the chromatin immunoprecipitation-sequencing (ChIP-seq) analysis showed that RORα-binding signals (Signals 1 to 4) were present on the regulatory regions of *Il6ra* (Fig. 5a)^24^. Additional ChIP analysis confirmed that RORα bound directly to Signal 3, which is located in the intronic region of *Il6ra*. In additional ChIP assays, binding of the acetylated H3 at lysine 9 (AcH3K9), a marker of transcriptional activation, was also decreased at Signal 3, which also suggests the RORα-induced repression of *Il6ra* (Fig. 5b). Finally, using a reporter gene encoding Signal 3, we confirmed that Signal 3 was repressed by RORα (Fig. 5c).

## Discussion

Here, we show for the first time that RORα is a key transcriptional factor that suppresses the IL-6-mediated hepatic APR by disrupting the IL-6Rα–STAT3 axis (Fig. 5d). Hepatocytes are the main source of the IL-6-mediated production of APPs. RORα, which is found at high level in the liver, may effectively modulate expression of these hepatokines^25,26^. In addition to inducing the secretion of APPs, the pathogenesis of ALF includes increased cellular oxidative stress and mitochondrial dysfunction^27,28^. In our previous studies, we demonstrated the protective effects of RORα on oxidative stress and mitochondrial dysfunction in the liver^19,29^. Also, RORα primes Kupffer cells to transform into M2 macrophages with anti-inflammatory properties^17^. Together with our previous data, our current findings show the inhibitory action of RORα in the broad spectrum of pathogenesis of ALF and suggest that RORα may be a potent therapeutic target for the treatment of ALF.

We found that RORα suppressed transcription of IL-6Rα by direct DNA binding and subsequent histone deacetylation at an intron region of IL-6Rα (Fig. 5). In general, RORα functions as an active transcriptional factor that promotes the recruitment of chromatin remodeling coactivators at target sites containing ROR response elements (ROREs)^30^. However, we and other researchers have reported that RORα also has a suppressive function on promoters of certain target genes through different modes of action. For example, RORα suppresses the expression of *Socs3* through direct RORE binding (classical pathway) but inhibits the transcription of *Cyp2e1* by binding to other transcription factors (nonclassical pathway)^31,32^. In the case of the *Il6ra* gene, no putative ROREs were found in the Signal 3 intron region of IL-6Rα. Instead, an *in silico* analysis suggested that binding sites for other transcription factors, such as the E2F transcription factor 1 and peroxisome proliferator-activated receptor γ, were present in this region (data not shown). Interestingly, these transcription factors were shown to induce transactivation or transrepression of downstream genes in a protein interaction-dependent manner^33^. Given that RORα represses *Cyp2e1* gene expression by a tethering transrepression mechanism via physical interaction with estrogen related receptor γ, a tethering mechanism similar to that of RORα with unrevealed transcription factor(s) might be involved in the transrepression of IL-6Rα^32^.

The pathogenesis of liver diseases includes multiple outcomes of the IL-6–STAT3 pathway including the APR, cancer cell proliferation, and regeneration^34^. Therefore, the development of therapeutic agents that target this pathway has been explored in recent decades. For example, STAT3 inhibitors such as AZD9150 and OPB-31121 are currently being evaluated in clinical trials for patients with hepatocellular carcinoma^9^. However, the clinical applications for these inhibitors remain elusive because their low binding specificity and rapid degradation cause low efficacy^35^. Tocilizumab, the first humanized IL-6R antibody approved by the US Food and Drug Administration, is approved for treatment of severe inflammatory disorders such as rheumatoid arthritis^36^. Tocilizumab reduces serum levels of APPs, including CRP, but it also increases serum levels of IL-6 and soluble IL-6Rα, and causes liver injury, thus the future of this drug’s use is under further consideration^37,38^. In this study, we show that the RORα activator JC1-40 inhibited the activation of the IL-6Rα–STAT3 axis and thereby decreased hepatic acute-phase inflammation without changing serum IL-6 level but decreased the transcription of IL-6Rα in mice (Figs 1 and 4). This effect of JC1-40 was abolished by knockdown of RORα, suggesting that it was mediated by RORα (Fig. 2c,d). Moreover, JC1-40 induced the transcriptional activity RORα, but not that of RORβ and RORγ (Supplementary Fig. S3). The ligand binding domain of RORα shows amino acid sequence homology of 63% and 58% with that of RORβ and RORγ, respectively, which may cause this differential ligand specificity^39^. Our results suggest that RORα activators could provide a good strategy for the development of effective therapeutics for liver diseases mediated by the IL-6Rα–STAT3 axis, such as hepatic injury with APR and hepatocellular carcinoma.

## Methods

### Cell culture and reagents

Primary mouse hepatocytes were isolated from 8–10-week-old male C57BL/6N mice (Orient Bio, Seongnam, Korea) as previously described^33^. The cells were plated in collagen-coated plates and maintained under 5% CO_2_ at 37° in Medium 199/Earle’s balanced salt solution (HyClone, Logan, UT, USA) supplemented with 10% fetal bovine serum (FBS). HepG2 cells were obtained from American Type Culture Collection (ATCC, Manassas, Virginia, USA) and maintained in Dulbecco’s modified Eagle’s medium supplemented with 10% FBS (HyClone). Mouse IL-6 was purchased from R&D Systems (Minneapolis, MN, USA), and human IL-6 was obtained from PeproTech (Rocky Hill, NJ, USA). SR1078 and S3I-201 were purchased from Tocris Bioscience (Bristol, UK) and Sigma-Aldrich (St. Louis, MO, USA), respectively. JC1-40 was synthesized and prepared as previously described^18,19,40^. Specificity of JC1-40 as a RORα ligand was described previously^19,32^.

### Recombinant adenovirus, siRNA, and reporter gene assay

Human RORα recombinant adenovirus, Ad-RORα, and control virus, Ad-GFP, and infection of these viruses were used as described previously^18^. The siRNA duplex targeting mouse RORα was synthesized by Samchully Pharm Co., Ltd (Supplementary Table S1) (Seongnam, Korea). Transient transfection of siRNA was performed using X-tremeGENE HP DNA transfection reagent (Roche, Mannheim, Germany) or Lipofectamine 2000 (Invitrogen, Carlsbad, CA, USA) as previously described^21^.

For reporter gene assays for pSTAT3 activity, HepG2 cells were transfected with a DNA mixture containing the Cignal STAT3 reporter plasmid (CCS-9028L; QIAGEN, Hilden, Germany) and β-galactosidase expression vector using Lipofectamine 2000 (Invitrogen) according to the manufacturer’s protocol. The pGL2 signal 3-luc construct contained the 172 bp fragment of *Il6ra* genomic loci amplified from mouse genomic DNA (Chromosome 3: 89,910,237–89,910,408) in a pGL2-promoter vector (Promega, Madison, WI, USA). The luciferase activity was normalized by β-galactosidase activity for transfection efficiency.

### Western blotting and real-time PCR

Western blotting was performed as described previously using specific antibodies against RORα, actin (Santa Cruz Biotechnology, Santa Cruz, CA, USA), pSTAT3, STAT3, JAK1, JAK2, SRC, PIAS3, and SHP-1 (Cell Signaling Technology, Beverly, MA, USA)^32^. Quantitative real-time PCR (qPCR) was performed using an ABI StepOnePlus^TM^ Real-time PCR system (Applied Biosystems, Foster City, CA, USA) using specific primers (Supplementary Table S1). Relative mRNA levels of target genes were estimated using the equation 2^−ΔCt^ (ΔCt = Ct of target gene minus Ct of β-actin or 18 S rRNA) and are presented relative to the level of the control group, which was designated as 1. The detailed method for qPCR is described in Han *et al*.^32^.

### Animal experiments and immunohistochemistry

Six-to-eight-week-old male C57BL/6N mice were obtained from Orient Bio Inc. (Seongnam, Korea), and housed in an air-conditioned room at 22–24 °C and 50–60% humidity with a 12 h light/dark cycle. JC1-40 was administered at a dose of 20 mg/kg/day in 0.5% carboxymethyl cellulose by oral gavage for 3 days, and mice were then injected with 100 mg/kg DEN (Sigma-Aldrich, St. Louis, MO, USA) by intraperitoneal injection. Two days after DEN treatment, the mice were sacrificed, liver tissues were excised rapidly, and portions of the liver were stored for further analysis of protein and mRNA or fixed in 10% formalin for histopathological analysis. Animal experiments were approved and conducted in accordance with guidelines of Seoul National University Animal Care and Use Committee (permission number SNU-130305-1).

For histological examination, 3 μm sections of paraffin-embedded tissues were stained with hematoxylin and eosin (H&E). Immunohistochemistry was performed using anti-IL-6Rα (Santa Cruz Biotechnology), anti-CXCL1 (Novus Biologicals, Littleton, CO, USA), anti-LCN2 (R&D Systems) anti-γH2AX (Abcam, Cambridge, MA, USA) antibodies. Serum concentrations of IL-6, TNFα, and IL-1β were measured using commercial ELISA kits (AbFrontier, Seoul, Korea) according to the manufacturer’s protocol.

### ChIP-seq and ChIP analysis

The binding signals of RORα on the *Il6ra* region were identified in the Gene Expression Omnibus database (GSE59486 for RORα ChIP-seq and GSE26345 for liver input control) according to the protocol described previously^24,29,41^. The ChIP assay was conducted using anti-RORα (Santa Cruz Biotechnology), anti-AcH3K9 (Abcam) antibodies, or a control IgG antibody (Santa Cruz Biotechnology). The immunoprecipitated genome region was amplified by SYBR Green Master mix (Applied Biosystems) with specific primers (Supplementary Table S1). Data were normalized to input and analyzed relative to the nonspecific IgG control.

### Statistics

All values are expressed as mean ± SD. The data were analyzed using the nonparametric Mann–Whitney *U* test for simple comparisons or Kruskal–Wallis ANOVA for multiple comparisons. *P* < 0.05 was considered to be significant.

## Supplementary information

Supplementary information
