# Supplementary material for: RORα suppresses interleukin-6-mediated hepatic acute phase response
Source: Sci Rep. 2019 Aug 13;9:11798. doi: 10.1038/s41598-019-48171-8 (PMC6692401; doi:10.1038/s41598-019-48171-8)
Supplement: Supplementary file 1 — Supplementary information [file 41598_2019_48171_MOESM1_ESM.docx]

**Supplementary Information**

**RORα suppresses interleukin-6-mediated hepatic acute phase response**

Ju-Yeon Kim^*^,Yong-Hyun Han^*^, Min-Woo Nam, Hyeon-Ji Kim, and Mi-OckLee^a^

From the College of Pharmacy and Bio-MAX institute, Research Institute of Pharmaceutical Sciences, Seoul National University, Seoul 08826, Republic of Korea

^a^Correspondence: molee@snu.ac.kr

*These authors contributed equally to this work.

*Address reprint requests to:*

Mi-Ock Lee Ph. D., College of Pharmacy, Seoul National University, San 56-1 Sillim-dong, Gwanak-gu, Seoul 08826, Republic of Korea. E-mail: [molee@snu.ac.kr](mailto:molee@snu.ac.kr). Phone: 82-2-880-9331

**Supplementary Figure**

**
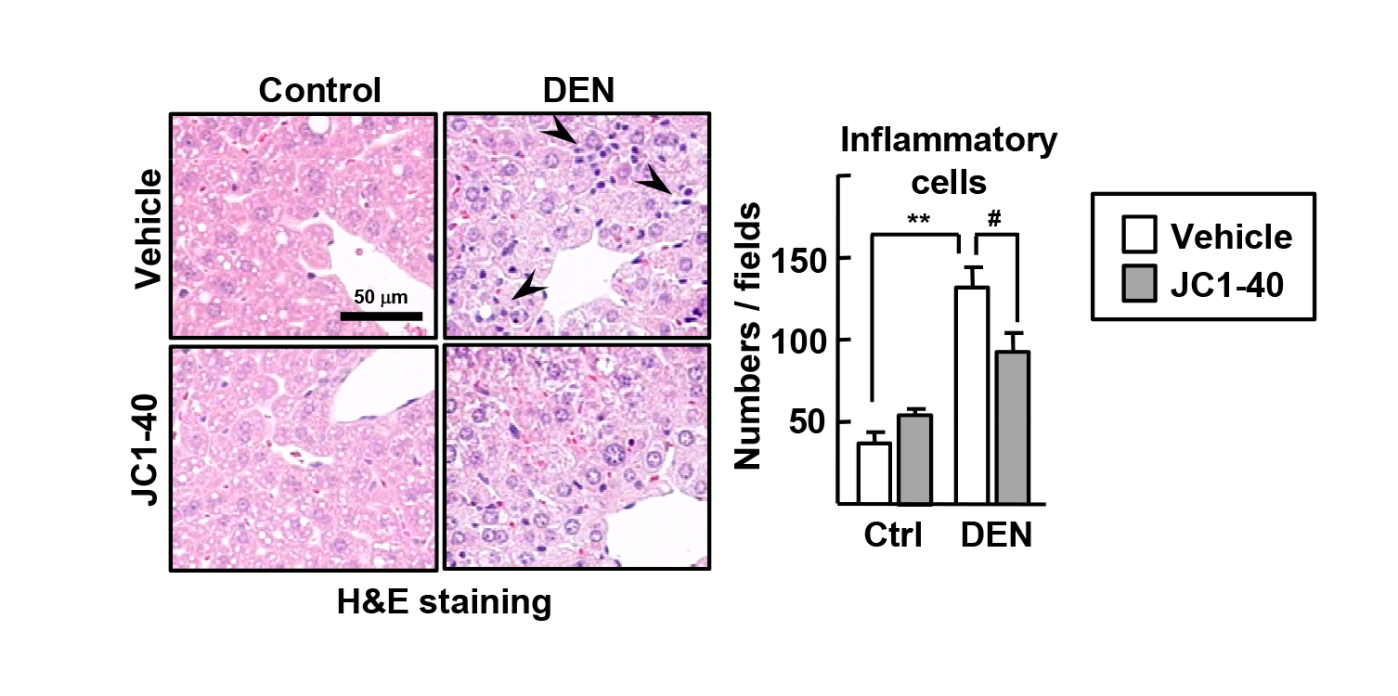
**

**Supplementary Figure S1. Administration of JC1-40 reduces DEN-induced infiltration of leukocytes in the mouse liver.**

JC1-40, 20 mg/kg BW/day, was administered orally for 3 days to the mice and then the mice were i.p. injected with 100 mg/kg BW DEN for 2 days before sacrificed. Representative H&E staining of liver sections. Arrow heads indicate infiltrated leukocytes around vein region (left). The values are number of infiltrated leukocytes in each fields of X200 magnification (right). ***P* < 0.01; ^#^*P* < 0.05 (n=5). The data represent mean±SD.

**
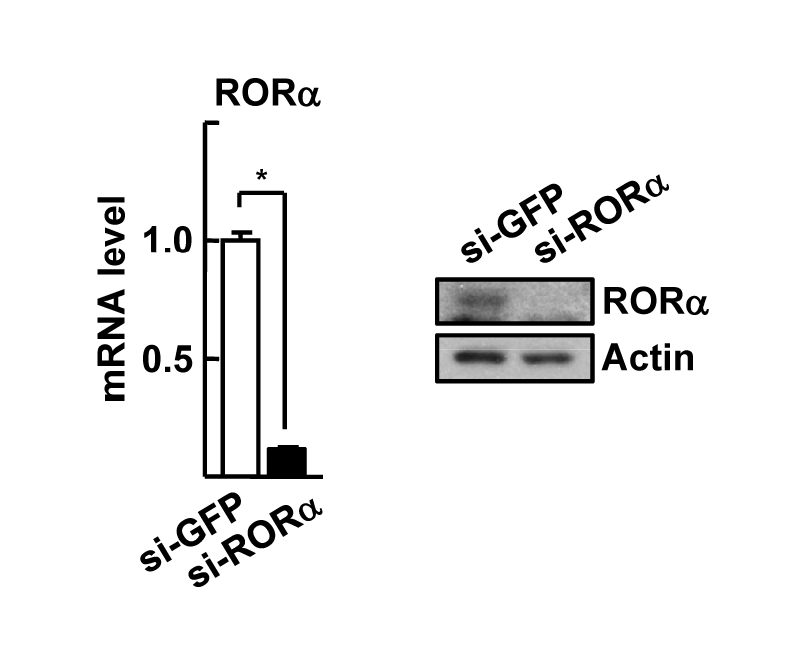
**

**Supplementary Figure S2. Validation of knock down of RORα expression after si-RNA treatment.**

Primary mouse hepatocytes were transfected with si-RORα or si-GFP for 48 h. The hepatic mRNA level of RORα was analyzed by qRT-PCR (left). The protein level of RORα in the hepatocytes was analyzed by western blotting (right). **P* < 0.05 (n=3). The data represent mean±SD.

**Supplementary Figure S3. Effect of JC1-40 on the transcriptional activity of ROR subfamily members.**

Chang cells were transfected with the Gal4-*tk*-Luc reporter with pM-RORα, pM-RORβ, or pM-RORγ and then treated with 20 μM or 30 μM JC1-40 for 24 h. Luciferase activity was measured and normalized by β-galactosidase activity. **P* < 0.05 (n=3). The data represent mean ± S.D.

**Supplementary Figure S4. Expression pattern of APP genes after knock down of RORα expressioin.**

Primary mouse hepatocytes were transfected with si-GFP or si-ROR**α** for 24 h and then treated with 20 ng/ml IL-6 for an additional 24 h. The mRNA levels of APR genes such as Cxcl1 and C3 were analyzed by qRT-PCR. **P* < 0.05; ^#^*P* < 0.05 (n=3). The data represent mean±SD.

**
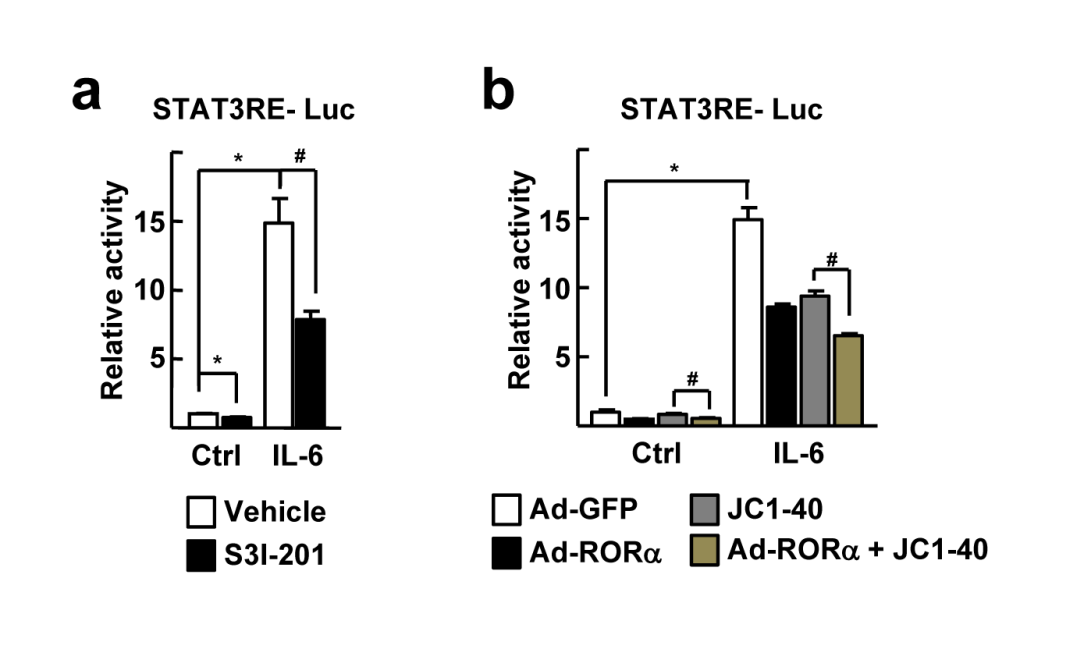
**

**Supplementary Figure S5. Regulation of STAT3 activity by overexpression of RORα.**

(**a**) HepG2 cells were transfected with the STAT3RE-Luc reporter. After 24 h of transfection, cells were and treated with 50 μM S3I-201 for 24 h in the presence or absence of 1 ng/ml IL-6 for 6 h. (**b**) HepG2 cells were transfected with the STAT3RE-Luc reporter. After 24 h of transfection, cells were infected by Ad-GFP or Ad-RORα and treated with 50 μM JC1-40 for 24 h in the presence or absence of 1 ng/ml IL-6 for 6 h. Luciferase activities were normalized by corresponding β-galactosidase activity (n=3). **P* < 0.05; ^#^*P* < 0.05 (n=3). The data represent mean±SD.

**Supplementary Table**

**Supplementary Table S1.** Oligonucleotide sequences used in the present investigation.

| **Gene** | **Accession number** | **Nucleotide sequence** | | **Species** | **Purpose** |
| --- | --- | --- | --- | --- | --- |
| Cxcl1 | NM_008176 | Sense  Antisense | 5'- CTGGGATTCACCTCAAGAACATC -3'  5'- CAGGGTCAAGGCAAGCCTC -3' | Mouse | qRT-PCR |
| Pai1 | NM_008871 | Sense  Antisense | 5'- TTCAGCCCTTGCTTGCCTC-3'  5'- ACACTTTTACTCCGAAGTCGGT-3' | Mouse |  |
| Lcn2 | NM_008491 | Sense  Antisense | 5'- TGCCACTCCATCTTTCCTGTT -3'  5'- GGGAGTGCTGGCCAAATAAG -3' | Mouse |  |
| SerpinA3N | NM_009252 | Sense  Antisense | 5'- CAATGTCTGCGAAACTGTACC -3'  5'- TTTGGGGTTGGCTATCTTGGC -3' | Mouse |  |
| C3 | NM_009778 | Sense  Antisense | 5'- AACTGCTGGCCTCTGGAGTA -3'  5'- GCATGATTCCTCGAGGTTGT -3' | Mouse |  |
| Mcp1 | NM_011333 | Sense  Antisense | 5'- CACTCACCTGCTGCTACTCA -3'  5'- GCTTGGTGACAAAAACTACAGC -3' | Mouse |  |
| Il6ra | NM_010559  NM_001310676 | Sense  Antisense | 5'- ATCCTCTGGAACCCCACAC -3'  5'- GAACTTTCGTACTGATCCTCGTG -3' | Mouse |  |
| Jak1 | NM_146145 | Sense  Antisense | 5'- GTCCCTGAAGCCTGAGAGTG -3'  5'- CTTGATACCATTGCCTCCGT -3' | Mouse |  |
| Jak2 | NM_008413  NM_001048177 | Sense  Antisense | 5'- GATGGCGGTGTTAGACATGA -3'  5'- TGCTGAATGAATCTGCGAAA -3' | Mouse |  |
| Pias3 | NM_146135 | Sense  Antisense | 5'- GATCCGGAATCCAGACCATTC -3'  5'- ACATGAGTGACACCCGGAGACT -3' | Mouse |  |
| Ptprd | NM_011211 | Sense  Antisense | 5'- TTGCACTTTATAAGAATGGATCGAGT -3'  5'- TTCTGCCAACACCAGCACTGCAGTGT -3' | Mouse |  |
| Shp1 | NM_013545 | Sense  Antisense | 5'- GACCTGGTGGAGCACTTCAAG -3'  5'- TCCTCCGACTCCTGCTTCTTG -3' | Mouse |  |
| Src | NM_009271 | Sense  Antisense | 5'- TCCACACCTCTCCGAAGCAA -3'  5'- CATGCTGATGGCCTGTGTCA -3' | Mouse |  |
| Abl1 | NM_001112703 | Sense  Antisense | 5'- TCGTTACCTCCAAAGGCTGCTC -3'  5'- ATGGCGGTGTCTGGCTATTCA -3' | Mouse |  |
| Socs3 | NM_007707 | Sense  Antisense | 5'- GCTCCAAAAGCGAGTACCAGC -3'  5'- AGTAGAATCCGCTCTCCTGCAG -3' | Mouse |  |
| 18S rRNA | NR_003278 | Sense  Antisense | 5'- GTAACCCGTTGAACCCCATT -3'  5'- CCATCCAATCGGTAGTAGCG-3' | Mouse |  |
| β-actin | NM_007393 | Sense  Antisense | 5'- CGTGGGCCGCCCTAGGCACCA -3'  5'- TGGCCTTAGGGTTCAGGGGGG-3' | Mouse |  |
| RORα | NM_013646  NM_001289916 |  | 5′-GCAGAGAGACAGCUUGUACGC-3′ | Mouse | si-RNA |
| IL-6Rα  Signal 1 |  | Sense  Antisense | 5'- GGCTTTCTGGAACCAGACTTTA -3'  5'- ACATTCTCTCTCTGGGACACT -3' | Mouse | ChIP |
| IL-6Rα  Signal 2 |  | Sense  Antisense | 5'- CTGACTCTGTCAAATACAAGGTAAA -3'  5'- TTCTCAGACTAATGGCTCCTG-3' | Mouse |  |
| IL-6Rα  Signal 3 |  | Sense  Antisense | 5'- CTTAGAGGTCTTTGCCTCCTG -3'  5'- CACTCTCTGCTCTTCTGATACAT -3' | Mouse |  |
| IL-6Rα  Signal 4 |  | Sense  Antisense | 5'- TGTGGTTCAGAAAGGACACC -3'  5'- TCTAAACTGGCAGGGCTTTC -3' | Mouse |  |

**Full-length gels and blots for figures**
